# Perilipin 5 Protects against Cellular Oxidative Stress by Enhancing Mitochondrial Function in HepG2 Cells

**DOI:** 10.3390/cells8101241

**Published:** 2019-10-11

**Authors:** Yanjie Tan, Yi Jin, Qian Wang, Jin Huang, Xiang Wu, Zhuqing Ren

**Affiliations:** 1 Key Laboratory of Agriculture Animal Genetics, Breeding and Reproduction of the Ministry of Education, College of Animal Science, Huazhong Agricultural University, Wuhan 430070, China; tanyanjie@webmail.hzau.edu.cn (Y.T.); hyj_1900@webmail.hzau.edu.cn (Y.J.); wangqian58@webmail.hzau.edu.cn (Q.W.); 13164367031@163.com (J.H.); wx1078724218@163.com (X.W.); 2 The Cooperative Innovation Center for Sustainable Pig Production, Huazhong Agricultural University, Wuhan 430070, China

**Keywords:** perilipin 5, lipid droplet, mitochondria, ROS

## Abstract

Non-alcoholic fatty liver disease (NAFLD) is one of the most common liver diseases worldwide. Reactive oxygen species (ROS), as potent oxidants in cells, have been shown to promote the development of NAFLD. Previous studies reported that for ROS-induced cellular oxidative stress, promoting lipid droplet (LD) accumulation is associated with the cellular antioxidation process. However, the regulatory role of LDs in relieving cellular oxidative stress is poorly understood. Here, we showed that *Perilipin 5* (*PLIN5*), a key LD protein related to mitochondria–LD contact, reduced ROS levels and improved mitochondrial function in HepG2 cells. Both mRNA and protein levels of *PLIN5* were significantly increased in cells with hydrogen peroxide or lipopolysaccharide (LPS) treatment (*p* < 0.05). Additionally, the overexpression of *PLIN5* promoted LD formation and mitochondria–LD contact, reduced cellular ROS levels and up-regulated mitochondrial function-related genes such as *COX* and *CS*. Knockdown *PLIN5*, meanwhile, showed opposite effects. Furthermore, we identified that cellular oxidative stress up-regulated *PLIN5* expression via the JNK-p38-ATF pathway. This study shows that the up-regulation of *PLIN5* is a kind of survival strategy for cells in response to stress. *PLIN5* can be a potential therapeutic target in NAFLD.

## 1. Introduction

There are a large number of patients suffering from non-alcoholic fatty liver disease (NAFLD) all over the world. This disease increases the risk of non-alcoholic hepatitis (NASH), chronic interstitial hepatitis, hepatic failure, and even hepatocellular carcinoma [1,2,3]. One of the characterizations of NAFLD is increased levels of reactive oxygen species (ROS) [4]. Some studies have shown that high levels of ROS promote the development of NAFLD/NASH and hepatocellular carcinoma by inducing ER stress [5] or regulating the AMPK signaling pathway [6]. There are several antioxidant enzymes such as superoxide dismutase (*SOD*), catalase (*CAT*), and glutathione peroxidase (*GPX*) responsible for scavenging cellular ROS. Meanwhile, the expression levels of *SOD* and other antioxidant enzymes are decreased in NAFLD/NASH [4]. Interestingly, lipid droplets (LDs) have been shown to be involved in the cellular stress response process. Bailey et al. demonstrated that lipid droplets can act as antioxidant organelles that protect *Drosophila* neural stem cells from hypoxia-triggered ROS [7], by allowing neuronal stem cells to keep proliferating under hypoxic conditions, and protection likely involves sequestering vulnerable membrane lipids away from ROS [8]. Furthermore, LDs also respond to starvation-induced stress by increasing their contact with mitochondria and lysosomes, which could consist in the role of these contacts in transferring fatty acids from LDs to mitochondria or lysosomes for energy supply [9]. Moreover, the formation of nuclear LDs is related to the stress induced by phospholipid shortages [10,11]. Our previous study has shown that hydrogen peroxide promoted the formation of cellular LDs [12]. However, whether the increased cellular LDs play a role as anti-oxidants is largely unknown.

*Perilipin 5* (*PLIN5*) is one of the conserved LD proteins, which belongs to the PAT (*perilipin, adipophilin,* and *TIP47*) protein family [13]. Oxidative tissues such as skeletal muscle, liver, and brown fat have high expression levels of *PLIN5*, indicating that *PLIN5* plays an important role in lipid storage and LD function [14,15,16]. Previous studies identified that *PLIN5* regulated triglyceride contents in hepatocytes [17] and skeletal muscle [18]. Overexpression of *PLIN5* in skeletal muscle promotes oxidative gene expression and lipid content [19]. Recently, *PLIN5* was reported to be the key factor that regulated LD contacting mitochondria [20,21]. The N-terminal (1-188aa) of *PLIN5* is the conserved PAT domain, and 189-391aa is the domain contacting with patatin like phospholipase domain containing 2 (*PNPLA2, ATGL*). The C-terminal of *PLIN5* (443-463aa) is the key sequence related to mitochondrial recruiting [22]. LD–mitochondria contact is important for the energy supply during starvation stress, which promotes lipid β-oxidation [9,23], and the transfer process of fatty acids from LDs to mitochondria was also observed by probe imaging [24,25]. Recently, a study also found that LD–mitochondria contact contributed to lipid synthesis and LD expansion [22]. Furthermore, LDs are able to protect against cellular apoptosis by clearing harmful proteins from outer mitochondrial membranes [26]. Moreover, *PLIN5* has been shown to limit fatty acid toxicity [27]. These studies suggested that *PLIN5* was involved in the process of cellular anti-oxidation.

In the present study, we found that hydrogen peroxide- or lipopolysaccharide (LPS)-induced oxidative stress up-regulated both mRNA and protein levels of *PLIN5*. The overexpression of *PLIN5* increased the cellular LD content, promoted LD–mitochondria contact, reduced cellular ROS level, and enhanced mitochondrial function-related gene expression, whereas knockdown *PLIN5* indicated opposite phenotypes. Moreover, we identified that the promoter region of *PLIN5* contained the binding sites of *JUN, ATF1, ATF3,* and *ATF4*, and therefore *PLIN5* expression was activated by the JNK-p38-ATF pathway. By bioinformatic analysis, it has been found that *PLIN5* has a high expression in liver hepatocellular carcinoma (LIHC), and additionally, low expression of *PLIN5* is correlated with poor prognosis in LIHC. Therefore, *PLIN5* can be a potential therapeutic target in NAFLD and NAFLD-induced LIHC.

## 2. Results

### 2.1. PLIN5 Was Up-Regulated in Liver Tissues of NAFLD Mice

**Figure 1:**
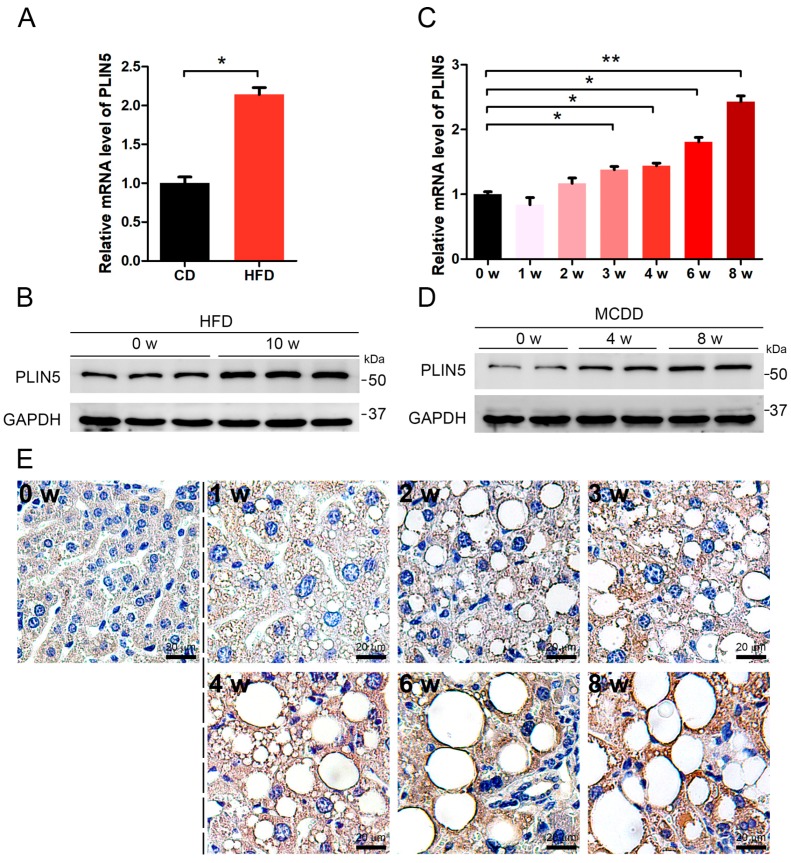
*Perilipin 5* (*PLIN5*) was up-regulated in liver tissues of non-alcoholic fatty liver disease (NAFLD) mice. (**A**) Six-week-old C57/bl male mice were fed with a high-fat diet (HFD) or chow diet (CD) for 10 weeks. The mRNA levels of *PLIN5* in liver tissues of mice fed with chow diet (CD) and HFD were detected by qRT-PCR. (**B**) The protein levels of *PLIN5* in liver tissues of mice fed with chow diet (CD) and HFD were detected by Western Blot. (**C**) Six-week-old C57/bl male mice were fed with methionine-choline-deficient diet (MCDD) for 1 week, 2 weeks, 3 weeks, 4 weeks, 6 weeks, and 8 weeks, respectively. The mRNA levels of *PLIN5* were detected by qRT-PCR. (**D**) The protein levels of *PLIN5* were detected by Western Blot. (**E**) Immunohistochemistry analysis of liver tissues of mice fed with MCDD (1–8 weeks) and control mice (0 week). Scale bar, 20 μm. These experiments were performed in triplicate. * *p* < 0.05; and ** *p* < 0.01.

NAFLD is characterized by the accumulation of LDs and a raised ROS level. We induced NAFLD in mice by two classical methods, which were the methionine-choline-deficient diet (MCDD) treatment and high-fat diet (HFD) treatment, respectively. The liver tissues of mice fed with MCDD for 0 week, 1 week, 2 weeks, 3 weeks, 4 weeks, 6 weeks, and 8 weeks, and mice fed with HFD for 0 week and 10 weeks were collected. Then, the changes in hepatic *PLIN5* expression were investigated in these collected samples. The results showed that the mRNA level of *PLIN5* was up-regulated significantly in hepatic tissues of mice fed with MCDD for 4 weeks, 6 weeks, and 8 weeks, and mice fed with HFD for 10 weeks, compared to the corresponding control samples (fed with chow diet, CD; *p* < 0.05; Figure 1A–D). To validate this phenotype and further investigate the localization of *PLIN5*, immunohistochemistry was performed. The hepatic tissues of mice fed with MCDD for 0 week, 1 week, 2 weeks, 3 weeks, 4 weeks, 6 weeks, and 8 weeks were detected. The results showed that *PLIN5* protein was mainly localized surrounding the LDs, and additionally, in mice fed with MCDD, the expression of *PLIN5* was activated (Figure 1E). These results indicate that the expression levels of *PLIN5* were enhanced and *PLIN5* was mainly recruited on the surface of LDs during NAFLD development.

### 2.2. Hydrogen Peroxide or LPS Treatment Enhanced Expression of PLIN5

**Figure 2:**
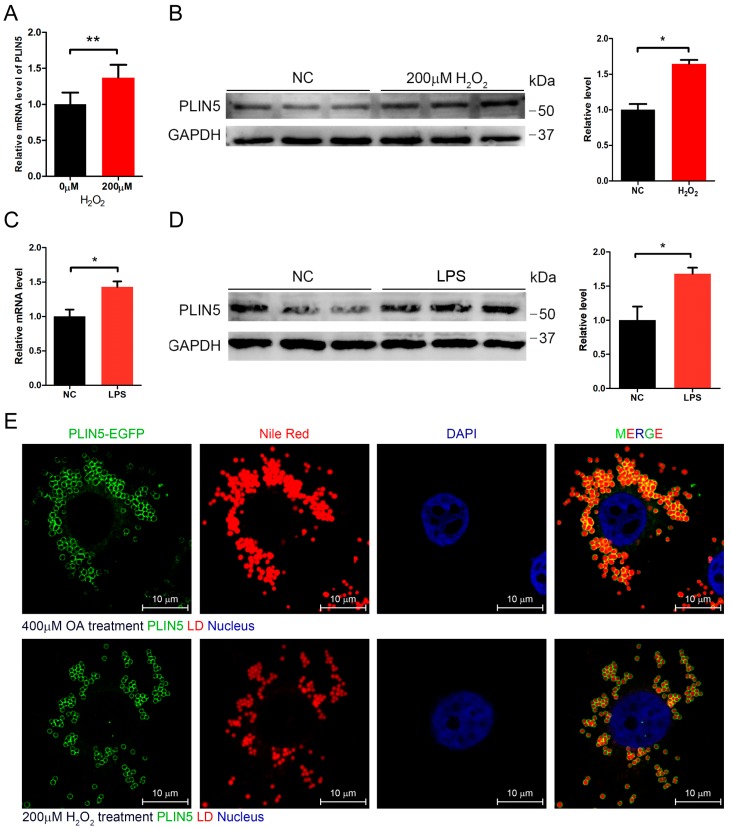
Hydrogen peroxide or lipopolysaccharide (LPS) treatment enhanced the expression of *PLIN5*. Hydrogen peroxide and LPS were used to induce cellular oxidative stress. (**A**) mRNA level of *PLIN5* in cells with H_2_O_2_ treatment or control (phosphate buffer saline, PBS). (**B**) Western Blot analysis of *PLIN5* protein levels in cells with H_2_O_2_ treatment or control (PBS). (**C**) mRNA level of *PLIN5* in cells with LPS treatment or control (PBS). (**D**) Western Blot analysis of *PLIN5* protein levels in cells with LPS treatment or control (PBS). (**E**) H_2_O_2_ treatment did not change the localization of *PLIN5*. *PLIN5* localization analysis of cells with 400 μM oleic acid medium or H_2_O_2_ treatment. Green, PLIN5-EGFP; red, lipid droplets; blue, nucleus. Bar, 10 μm. These experiments were performed in triplicate. * *p* < 0.05; and ** *p* < 0.01.

It was already well known that ROS levels were increased in hepatic tissues with NAFLD. Moreover, we did not observe any significant up-regulation of hepatic *PLIN5* in mice fed with MCDD for 1 week, 2 weeks, and 3 weeks, although many hepatic LDs had accumulated. Therefore, we assumed that the raised ROS levels activated *PLIN5* expression. ROS represents a variety of molecules and free radicals (chemical species with one unpaired electron) derived from molecular oxygen. Superoxide anion (O_2_^−^•), the product of a one-electron reduction of oxygen, is the precursor of most ROS and a mediator in oxidative chain reactions [28], and then hydrogen peroxide arises from O_2_^−^• [29,30]. Hydrogen peroxide and LPS are classical regents that induce cellular oxidation. Therefore, we investigated whether inducing cellular oxidative stress affected the expression level of *PLIN5*. The HepG2 cells were treated with 200 μM hydrogen peroxide for 12 h. qRT-PCR showed that *PLIN5* mRNA was up-regulated significantly by hydrogen peroxide treatment (*p* < 0.05; Figure 2A), and, additionally, the Western Blot indicated that *PLIN5* protein was also significantly up-regulated (*p* < 0.05; Figure 2B). Furthermore, we also investigated the changes in *PLIN5* expression levels with the lipopolysaccharide (LPS) treatment. The results showed that both mRNA and protein levels of *PLIN5* were up-regulated significantly (*p* < 0.05; Figure 2C,D). The oleic acid (OA) treatment can induce cells to form more and larger LDs, which is well applicable to observe the subcellular localization of *PLIN5*. Therefore, we subsequently detected the subcellular localization of *PLIN5* in OA treated cells by the PLIN5-EGFP expression vector. The results indicated that *PLIN5* was located on the surface of LDs (Figure 2E). To investigate whether hydrogen peroxide treatment changes the localization of *PLIN5*, we detected subcellular localization of *PLIN5* in cells with the 200 μM hydrogen peroxide treatment. We found that hydrogen peroxide treatment did not change the localization of *PLIN5* (Figure 2E). These results indicated that increased cellular ROS levels promoted the expression of *PLIN5*.

### 2.3. PLIN5 Regulated Cellular ROS Levels

**Figure 3:**
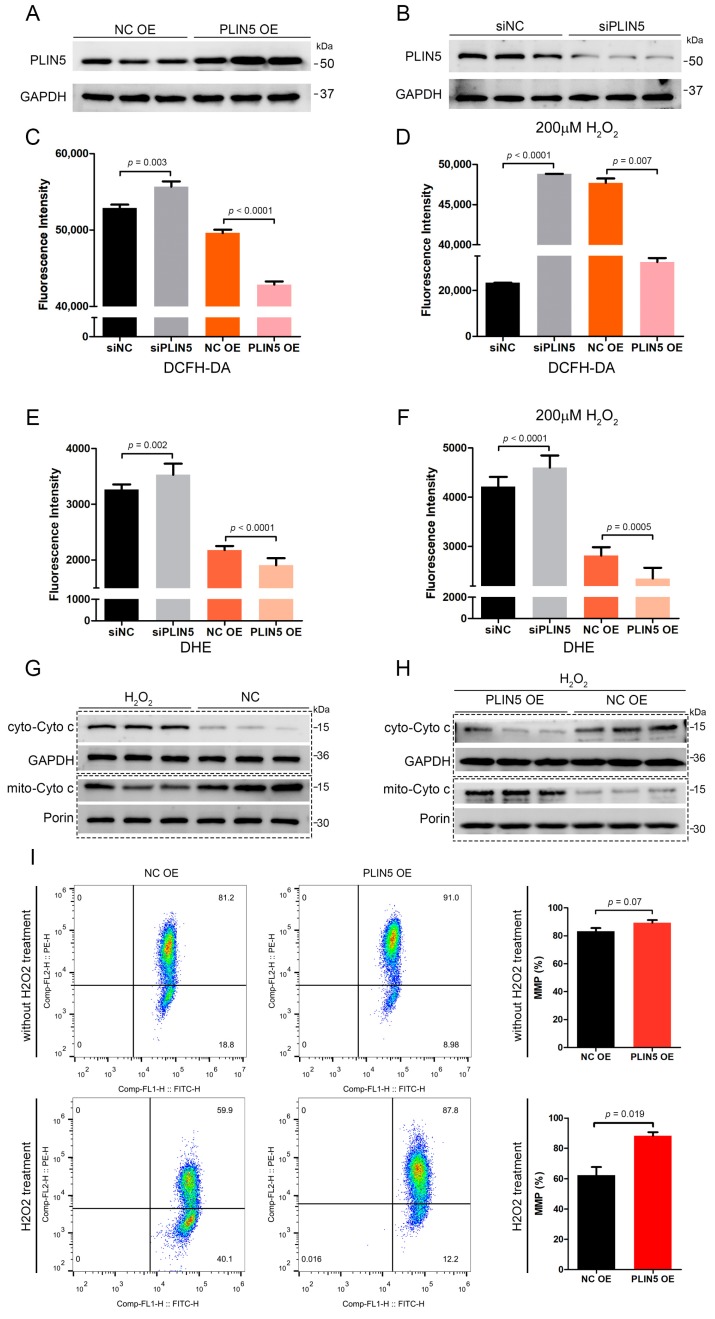
*PLIN5* regulated cellular reactive oxygen species (ROS) levels. (**A,B**) Western blot validation of *PLIN5* overexpression (**A**) and RNAi (**B**) efficiency. (**C**) HepG2 cells were transfected with *PLIN5* siRNAs or negative control siRNAs or *PLIN5* expression vector or pcDNA3.1 (control) vector, respectively. Then, the cellular ROS levels were detected by DCFH-DA probes through a microplate reader (Ex = 488 nm, Em = 525 nm). (**D**) The cells were transfected with *PLIN5* siRNAs or negative control siRNAs or *PLIN5* expression vector or pcDNA3.1 (control) vector, respectively, and then treated with 200 μM H_2_O_2_. Then, the cellular ROS levels were detected by DCFH-DA probes through a microplate reader (Ex = 488 nm, Em = 525 nm). (**E**) HepG2 cells were transfected with *PLIN5* siRNAs or negative control siRNAs or *PLIN5* expression vector or pcDNA3.1 (control) vector, respectively. Then, the cellular ROS levels were detected by DHE through a microplate reader (Ex = 535 nm, Em = 610 nm). (**F**) The cells were transfected with *PLIN5* siRNAs or negative control siRNAs or *PLIN5* expression vector or pcDNA3.1 (control) vector, respectively, and then treated with 200 μM H_2_O_2_. Then, the cellular ROS levels were detected by DCFH-DA probes through a microplate reader (Ex = 488 nm, Em = 525 nm). (**G**) The cells were treated with 200 μM H_2_O_2_. The cytoplasm and mitochondria were isolated respectively, and then cytosolic and mitochondrial cytochrome c levels were detected respectively through Western Blot. (**H**) The cells were transfected with *PLIN5* expression vector or pcDNA3.1 (control) vector, and then treated with 200 μM H_2_O_2_. The cytoplasm and mitochondria were isolated, and then cytosolic and mitochondrial cytochrome c levels were detected respectively through Western Blot. *GAPDH* was the reference protein of cytosolic component and the *Porin/VDAC1* was the reference protein of mitochondrial component. (**I**) The mitochondrial membrane potential (MMP) was detected by JC-1 probes using the flow cytometry method. These experiments were performed in triplicate. * *p* < 0.05. Ex, excitation wavelength; Em, emission wavelength.

To investigate whether *PLIN5* was involved in the anti-oxidant process, we validated the efficiency of *PLIN5* overexpression and knockdown by the Western Blot method first. The result showed that *PLIN5* was overexpressed and interfered successfully (Figure 3A,B). Subsequently, we knocked down and overexpressed *PLIN5* to detect ROS levels by the DCFH-DA (2,7-dichlorodihydrofluorescein diacetate) method, respectively. The results showed that *PLIN5* knockdown increased ROS levels, whereas *PLIN5* overexpression decreased ROS levels significantly (*p* < 0.05; Figure 3C). Subsequently, we used 200 μM hydrogen peroxide to treat cells with *PLIN5* knockdown and overexpression in order to investigate whether *PLIN5* expression affected ROS levels of cells in oxidative stress. The results indicated that *PLIN5* knockdown increased ROS levels, whereas *PLIN5* overexpression decreased ROS levels significantly in cells treated with 200 μM hydrogen peroxide (*p* < 0.05; Figure 3D). To validate the phenotype, we further used the DHE (dihydroethidium) method to detect the O_2_^−^• levels in *PLIN5* knockdown, overexpression, and corresponding control cells, respectively. The microplate reader indicated that *PLIN5* knockdown increased ROS levels, whereas *PLIN5* overexpression decreased ROS levels (Figure 3E,F). The release level of cytochrome c from mitochondria to cell plasma is the gold standard to reflect the level of cellular oxidative stress. Therefore, we investigated whether *PLIN5* knockdown aggravated or whether *PLIN5* overexpression reduced hydrogen peroxide-induced cytochrome c release. Firstly, we treated the cells with 200 μM hydrogen peroxide and then isolated the cytosolic and mitochondrial fractions respectively to detect the levels of cytochrome c (Figure 3G). The result showed that hydrogen peroxide treatment increased cytosolic cytochrome c levels and decreased mitochondrial cytochrome c levels, which indicated that hydrogen peroxide treatment increased cytochrome c releasing from mitochondria to cytoplasm. Then we detected the effect of *PLIN5* overexpression on the hydrogen peroxide treatment-induced cytochrome c release. The cells were transfected with *PLIN5* expression vector or pcDNA3.1 (control) vector, and then treated with 200 μM hydrogen peroxide. The cytoplasm and mitochondria were isolated, and then cytosolic and mitochondrial cytochrome c levels were detected through Western Blot, respectively. The result showed that *PLIN5* overexpression decreased cytosolic cytochrome c levels but increased mitochondrial cytochrome c levels compared to the control in the presence of hydrogen peroxide (Figure 3H). Mitochondrial membrane potential is an important indicator for mitochondrial oxidative damage. Therefore, we detected the mitochondrial membrane potential in cells with *PLIN5* overexpression or control by the JC-1 (5,5′,6,6′-tetrachloro-1,1′,3,3′-tetraethyl-imidacarbocyanine) method. As expected, the mitochondrial membrane potential was significantly higher in cells with *PLIN5* overexpression than in the corresponding control cells (Figure 3I). Furthermore, we detected the mitochondrial membrane potential in cells with *PLIN5* overexpression or control in the presence of hydrogen peroxide. The result showed that mitochondrial membrane potential of *PLIN5* overexpression group was higher than the control group (Figure 3I).

### 2.4. PLIN5 Promoted LD Formation and Contact with Mitochondria

**Figure 4:**
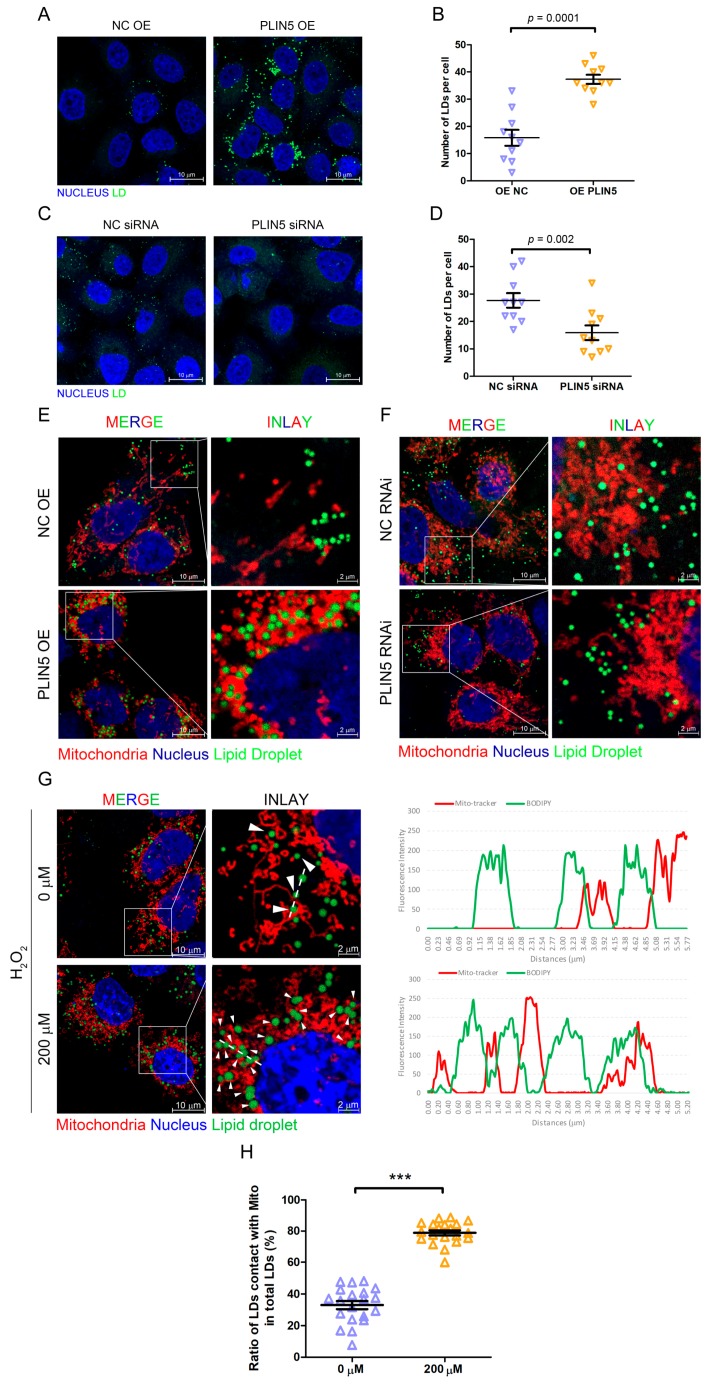
*PLIN5* promoted LD formation and contact with mitochondria. (**A**) HepG2 cells were transfected with *PLIN5* expression vector or pcDNA3.1 (control) vector. Then, the cellular lipid droplets were stained with BODIPY493/503 and observed by a confocal microscope. (**B**) The counts of cellular LDs in A. (**C**) HepG2 cells were transfected with *PLIN5* siRNAs or negative control siRNAs. Then, the cellular lipid droplets were stained with BODIPY493/503 and observed by a confocal microscope. (**D**) The counts of cellular LDs in C. (**E**) HepG2 cells were transfected with the mito-Dsred vector and *PLIN5* expression vector or pcDNA3.1 (control) vector. Then, the cellular lipid droplets were stained with BODIPY493/503 and observed by a confocal microscope. (**F**) HepG2 cells were transfected with mito-Dsred vector and *PLIN5* siRNAs or negative control siRNAs. Then, the cellular lipid droplets were stained with BODIPY493/503 and observed by a confocal microscope. (**G**) HepG2 cells were transfected with mito-Dsred vector and then treated with 200 μM H_2_O_2_. Then, the cellular lipid droplets were stained with BODIPY493/503 and observed by a confocal microscope. The fluorescence intensity along with the dotted line was performed to illustrate the contacts between LDs and mitochondria. (**H**) The ratio of contacts between LDs and mitochondria was analyzed. These experiments were performed in triplicate. *** *p* < 0.0001.

We have shown that up-regulated *PLIN5* decreased cellular ROS levels, so we then investigated the regulatory mechanism. Our previous study has shown that the up-regulation of *PLIN2* promoted the formation of cellular LDs [12]. Therefore, *PLIN5* was overexpressed in HepG2 cells, and then the cellular LDs were labeled by BODIPY493/503 (4,4-difluoro-1,3,5,7,8-pentamethyl-4-bora-3a,4a-diaza-s-indacene) to reflect the effect of *PLIN5* up-regulation on LD content. As expected, the number of cellular LDs in cells with *PLIN5* overexpression was higher than the LDs in control cells (*p* < 0.05; Figure 4A,B). To validate the phenotype, the LD content was detected in cells transfected with siRNA oligos targeting *PLIN5* and control cells. The result indicated that *PLIN5* knockdown decreased the number of cellular LDs (*p* < 0.05; Figure 4C,D). A previous study showed that *PLIN5* promoted LD contact with mitochondria, and subsequently, we validated this phenotype in HepG2 cells. There was no doubt that the result indicated that the overexpression of *PLIN5* highly enhanced this contact in HepG2 cells (*p* < 0.05; Figure 4E,F). Moreover, we also investigated whether hydrogen peroxide treatment induced LD contact with mitochondria, since the high levels of ROS up-regulated *PLIN5*. The mitochondria were labeled by a mito-tracker, and LDs were marked by BODIPY. After the treatment with hydrogen peroxide, the contact events were increased significantly compared to corresponding cells (*p* < 0.05; Figure 4G,H). The results indicated that high ROS levels enhanced LD formation and promoted LD contact with mitochondria by up-regulating *PLIN5*.

### 2.5. PLIN5 Regulated the Expression Levels of Mitochondrial Function-Related Genes

**Figure 5:**
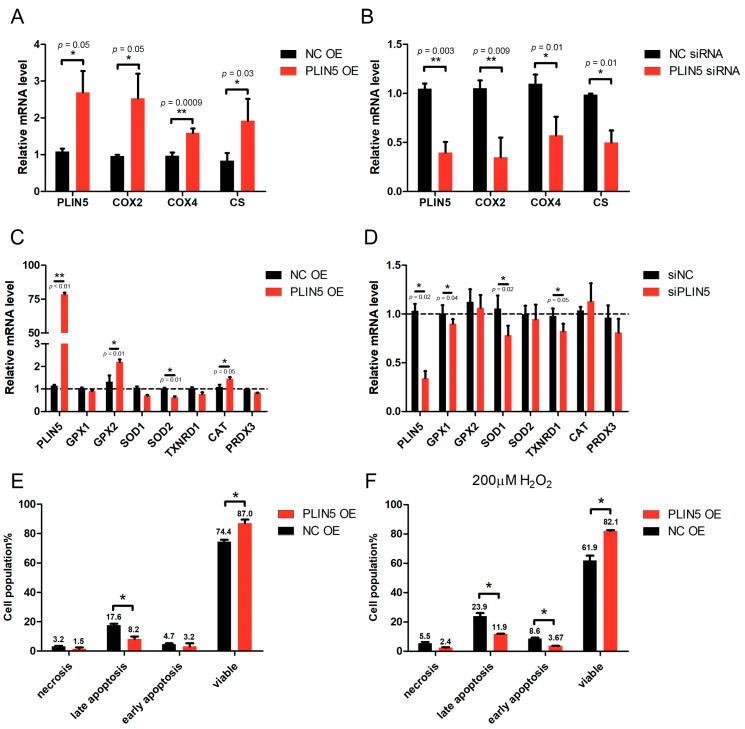
*PLIN5* regulated the expression levels of mitochondrial function-related genes and apoptosis rate. (**A**) mRNA levels of *COX2, COX4,* and *CS* in cells transfected with *PLIN5* expression vector or control vector. (**B**) mRNA levels of *COX2, COX4,* and *CS* in cells transfected with *PLIN5* siRNAs or control siRNAs. (**C**) mRNA levels of *GPX1, GPX2, SOD1, SOD2, TXNRD1, CAT,* and *PRDX3* in cells transfected with *PLIN5* expression vector or control vector. (**D**) mRNA levels of *GPX1, GPX2, SOD1, SOD2, TXNRD1, CAT,* and *PRDX3* in cells transfected with *PLIN5* siRNAs or control siRNAs. (**E**) Apoptosis rate of cells transfected with *PLIN5* expression vector or control vector. (**F**) Cells transfected with *PLIN5* expression vector or control vector were treated with 200 μM H_2_O_2_ for 12 h. Then, the apoptosis rate was analyzed. These experiments were performed in triplicate. *GAPDH* was used as the reference gene. * *p* < 0.05; and ** *p* < 0.01.

One of the functions of mitochondria is oxidative metabolism, which requires several mitochondrial respiratory chain oxidases, such as *COX* and *CS*. The expression levels of *COX* and *CS* are related to the mitochondria activity. Therefore, we further investigated whether PLIN5 expression affected the expression of *COX* and *CS*. The qPCR results showed that *COX2*, *COX4*, and *CS* were up-regulated in the cells with *PLIN5* overexpression (Figure 5A), whereas they were down-regulated significantly in cells with *PLIN5* knockdown (*p* < 0.05; Figure 5B). Subsequently, we investigated the effect of *PLIN5* expression on the expression levels of several cellular anti-oxidant genes including *GPX1, GPX2, SOD1, SOD2, TXNRD1, CAT,* and *PRDX3* through qPCR. The result showed that *GPX2* and *CAT* mRNA levels were increased significantly after *PLIN5* overexpression (*p* < 0.05), but *SOD2* mRNA level was decreased (Figure 5C). After *PLIN5* knockdown, the mRNA levels of *GPX1*, *SOD1*, and *TXNRD1* were decreased significantly (*p* < 0.05; Figure 5D).

### 2.6. PLIN5 Reduced Apoptotic Rates of HepG2 Cells

The mitochondrial activity is important for the cellular apoptosis process; therefore, we further investigated the effect of *PLIN5* on the regulation of cellular apoptosis. The rates of apoptosis of HepG2 cells were detected by the flow cytometry method. The results showed that *PLIN5* overexpression decreased the apoptotic rates significantly (*p* < 0.05; Figure 5E). Subsequently, a rescue experiment was carried out. *PLIN5* was overexpressed in the cells treated with hydrogen peroxide for 12 h, and then the apoptotic rates were detected. The result indicated that *PLIN5* overexpression rescued the enhancement of cellular apoptosis induced by hydrogen peroxide treatment (*p* < 0.05; Figure 5F).

### 2.7. The Expression of PLIN5 Was Regulated by the JNK-p38-ATF Pathway

**Figure 6:**
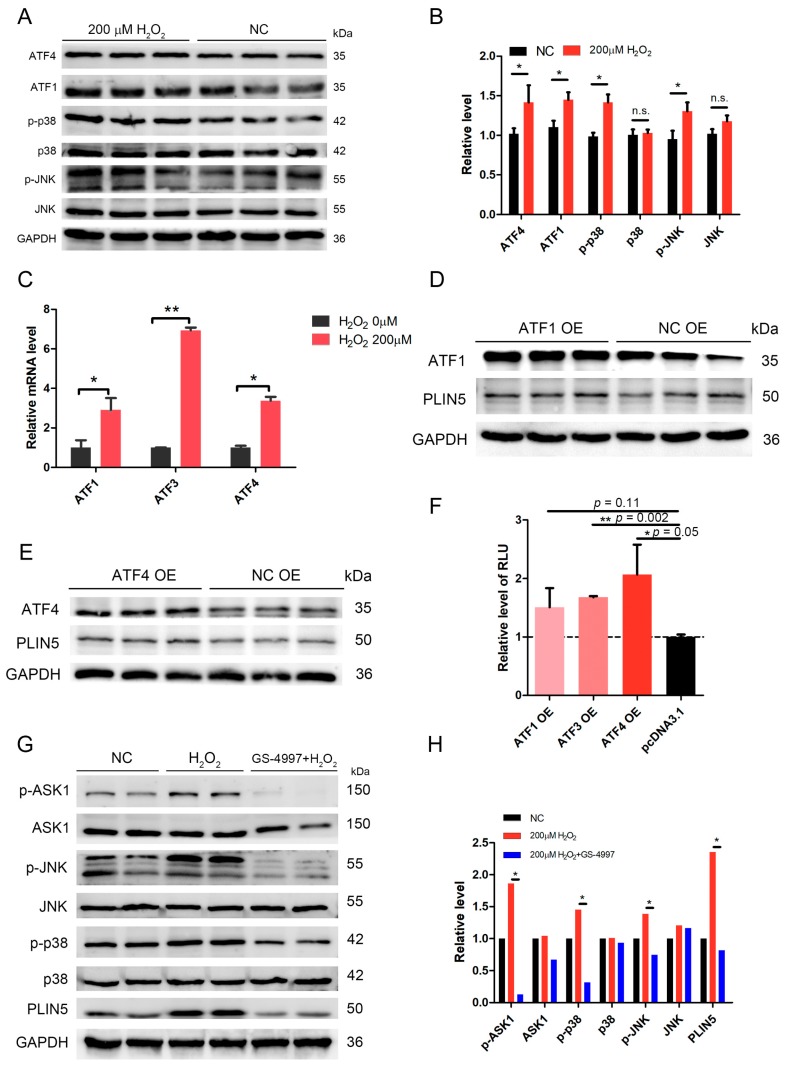
The expression of *PLIN5* was regulated by the JNK-p38-ATF pathway. (**A**) Protein levels of *ATF1, ATF4, p-p38, p38, p-JNK,* and *JNK* were detected by Western Blot. (**B**) The gray value analysis of A. (**C**) mRNA levels of *ATF1, ATF3,* and *ATF4* in cells with 200 μM H_2_O_2_ treatment were detected by qPCR. (**D**) The cells were transfected with *ATF1* expression vector or pcDNA3.1 vector. The protein levels of *ATF1* and *PLIN5* were detected through Western Blot. (**E**) The cells were transfected with *ATF4* expression vector or pcDNA3.1 vector. The protein levels of *ATF4* and *PLIN5* were detected through Western Blot. (**F**) The effects of ATFs’ expression on *PLIN5* transcriptional activity were detected by dual-luciferase reporter assay. (**G**) Protein levels of *p-ASK1, Ask1, p-p38, p38, p-JNK, JNK,* and *PLIN5* were detected by Western Blot. (**H**) The gray value analysis of G. *GAPDH* was used as the reference protein. These experiments were performed in triplicate. * *p* < 0.05; ** *p* < 0.01; and n. s., not significant.

We analyzed the promoter region to investigate the transcriptional regulation mechanism of *PLIN5* expression. The GeneHancer dataset showed two potential promoter/enhancer regions (GH19J004539 and GH19J004534) whose distances from TSS (transcription start site) were −5.3 kb and +0.1 kb. Then, the transcription factor binding sites in these two regions were analyzed. We found that these two regions contained *JNK, ATF1,* and *ATF4* binding sites. It is well known that JNK-p38 is an important signaling involved in stress response, which is activated by oxidative stress, DNA damage, and UV, and subsequently regulates the downstream targets’ expression, such as ATFs and STATs [31,32,33,34]. We have shown that *PLIN5* was up-regulated by hydrogen peroxide treatment; therefore, we assumed that *PLIN5* expression was regulated by the JNK-p38-ATF pathway. The phosphorylation levels of *p38* and *JNK* were detected. The Western Blot results indicated that *p-p38* and *p-JNK* levels were significantly increased in cells with hydrogen peroxide treatment (*p* < 0.05; Figure 6A,B). Moreover, the downstream targets of JNK-p38, *ATF1*, and *ATF4* were also up-regulated (Figure 6A,B). To further investigate whether ATFs regulate *PLIN5* expression, we overexpressed ATF1 and ATF4 and detected the *PLIN5* expression levels of both mRNA and protein. The qPCR and WB results showed that both mRNA and protein levels of *PLIN5* were increased significantly by either *ATF1* or *ATF4* overexpression (*p* < 0.05; Figure 6C–E). Subsequently, we cloned the promoter/enhancer regions of *PLIN5* (−2 kb) into pGL3-basic reporter vector to confirm the regulatory role of ATFs in *PLIN5* expression. The dual luciferase reporter gene assay showed that the fluorescence intensity of cells with *ATF1* or *ATF4* overexpression was much higher than that of the control cells (Figure 6F). The results indicated that both *ATF1* and *ATF4* did promote the transcriptional activity of *PLIN5*. To further validate the effect of JNK-p38 pathway on the expression of *PLIN5*, we utilized the p38-JNK pathway inhibitor, GS-4997 (Selonsertib). GS-4997 could inhibit the activity of *ASK1* so that to suppress the phosphorylation of downstream targets, *JNK* and *p38*. We found that hydrogen peroxide treatment activated the JNK-p38 pathway, but the GS-4997 treatment suppressed the JNK-p38 pathway (Figure 6G,H). Furthermore, we also found that hydrogen peroxide treatment increased the expression levels of *PLIN5*, and whereas GS-4997 treatment blocked the upregulation of *PLIN5* induced by hydrogen peroxide treatment (Figure 6G,H).

### 2.8. Low Expression of PLIN5 Is Associated with Poor Prognosis

**Figure 7:**
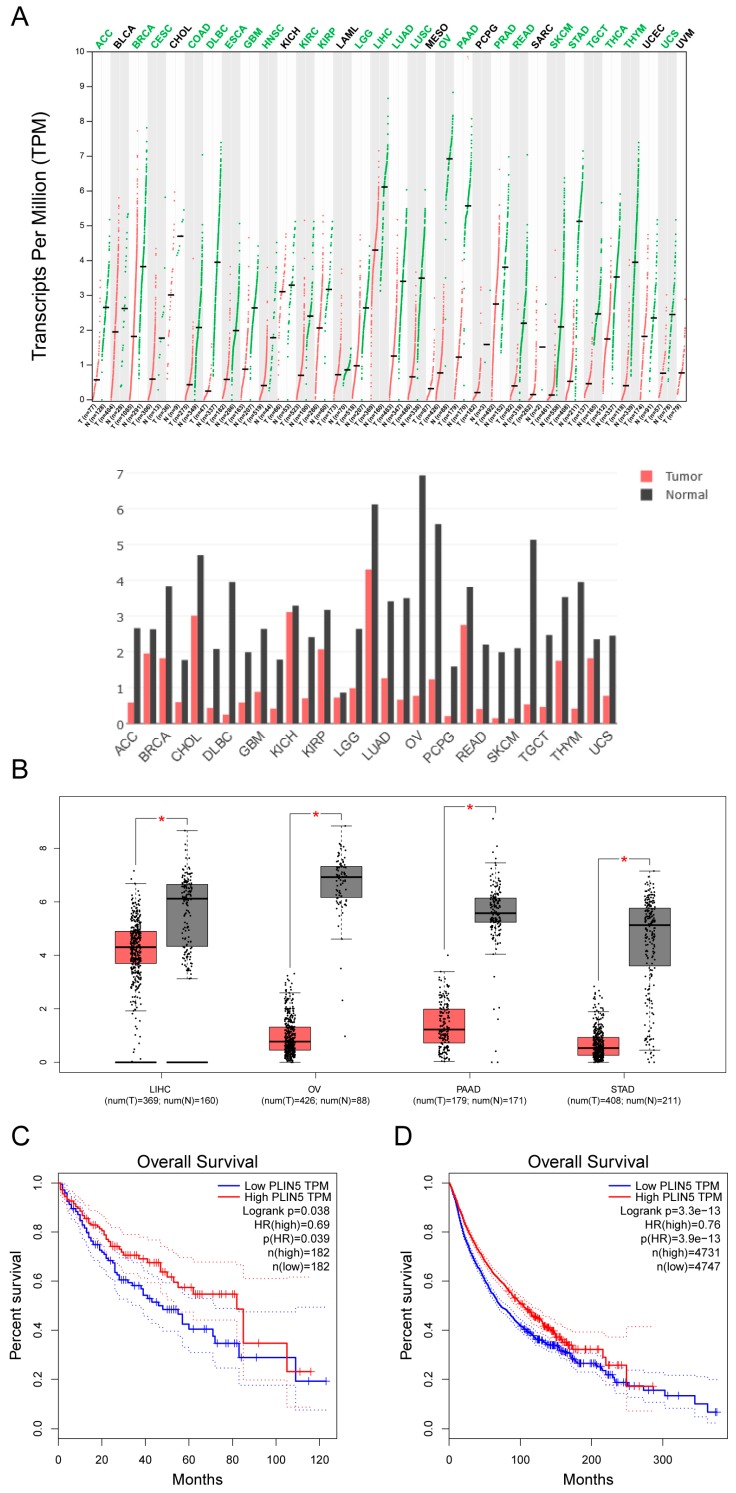
Low expression of *PLIN5* is associated with poor prognosis. Gene expression analysis and survival analysis were performed using the GEPIA database (http://gepia.cancer-pku.cn/). (**A**) Gene expression analysis of *PLIN5* by cancer type. The lower pattern is the histogram illustration of the upper pattern. (**B**) The expression levels of *PLIN5* in samples with LIHC, OV, PAAD, STAD, and corresponding controls, respectively. (**C**) Survival analysis of *PLIN5* in LIHCtumor samples. (**D**) Survival analysis of PLIN5 in different kinds of tumor samples including ACC, BLCA, BRCA, CESC, CHOL, COAD, DLBC, ESCA, GBM, HNSC, KICH, KIRC, KIRP, LAML, LGG, LICH, LUAD, LUSC, OV, PPAD, READ, SARC, SKCM, STAD, TGCT, THCA, THYM, UCEC and UCS; E, CESC, DLBC, HNSC, LIHC, LUSC, PPAD and THYM; F, ACC, BLCA, BRCA, CESC, CHOL, COAD, DLBC, ESCA, GBM, HNSC, KICH, KIRC, KIRP, LAML, LGG, LICH, LUAD, LUSC, OV, PPAD, READ, SARC, SKCM, STAD, TGCT, THCA, THYM, UCEC, and UCS. The extension of tumor abbreviations can be referred to in the GEPIA database. * *p* < 0.05.

We have shown that *PLIN5* expression was enhanced by oxidative stress and *PLIN5* could alleviate cellular ROS levels. We then analyzed the expression level of *PLIN5* in different kinds of tumors via the GEPIA (gene expression profiling interactive analysis) database (http://gepia.cancer-pku.cn/). Interestingly, many kinds of tumor samples showed lower *PLIN5* expression compared to normal samples (Figure 7A). Furthermore, among these kinds of tumor samples, liver hepatocellular carcinoma (LIHC), ovarian serous cystadenocarcinoma (OA), pancreatic adenocarcinoma (PAAD), and stomach adenocarcinoma (STAD) showed the largest differences (Figure 7B). Subsequently, survival analysis showed that the prognosis of LIHC was poor with a low expression level of *PLIN5* (Figure 7C). Then, survival analysis was performed to predicate the prognosis of 31 kinds of tumors with lower expressions of *PLIN5* (including ACC, BLCA, BRCA, CESC, CHOL, COAD, DLBC, ESCA, GBM, HNSC, KICH, KIRC, KIRP, LAML, LGG, LICH, LUAD, LUSC, OV, PPAD, READ, SARC, SKCM, STAD, TGCT, THCA, THYM, UCEC, and UCS; the extension of tumor abbreviations can be referred to in GEPIA). The result indicated that a low expression level of *PLIN5* was associated with poor prognosis (Figure 7D). These results indicated that low expression levels of *PLIN5* were bad for the prognosis of tumors.

## 3. Discussion and Conclusions

NAFLD has become one of the most common liver metabolic diseases worldwide. One obvious characterization of NALFD is an accumulation of LDs. A high level of hepatic fat accumulation increased cellular free fatty acids in hepatocytes, which was induced by lipolysis. However, the overaccumulation of free acids is dangerous because of toxic metabolites generated by fatty acid breakdown. Moreover, high lipid content showed higher levels of ROS [35]. Indeed, in liver tissues with NAFLD, a high level of oxidative damage was observed [4]. The enhanced oxidative stress resulted in changes in mitochondrial permeability transition, which was able to decrease the mitochondrial membrane potential and subsequently induce cell apoptosis. It is well known that decreasing the number of hepatocytes impairs the hepatic function and promotes NAFLD/NASH development [36]. Therefore, reducing cellular ROS levels contributed to the alleviation of oxidative damage, which was good for NAFLD/NASH treatment. However, the expression levels of SOD and other antioxidant enzymes, the main scavengers of cellular ROS, were decreased in NAFLD/NASH tissues [4]. Therefore, we considered that a compensation mechanism could exist to respond to this case.

*PLIN5* is a conserved LD protein that belongs to the PAT family [13]. *PLIN5* is expressed mainly in tissues with high oxidative metabolism such as liver, skeletal muscle, cardiac muscle, and brown adipose tissues. It is interesting that *PLIN5* was reported to be the key factor regulating LDs contacting mitochondria [37]. 443-463aa is the key region that promotes LDs’ recruitment to mitochondria [22]. The deletion of 443-463aa of *PLIN5* deprived the ability of *PLIN5* promoting LDs contacting mitochondria [22]. Additionally, 443-463aa region of *PLIN5* is highly conserved between different species [22]. A previous study has showed that the overexpression of *PLIN5* promoted cellular LD accumulation, whereas knockdown *PLIN5* enhanced fatty acid oxidation metabolism in liver cells [38]. Moreover, an SNP (single nucleotide polymorphism; rs327694326, NC_010444.4:g.74314701T>C) in Italy big white, Italy Duroc, and Peter ran pigs, which induced high expression levels of *PLIN5*, promoted lipid accumulation and decreased the levels of *HSL*, an important lipolysis. Ilan et al. overexpressed *PLIN5* in mouse brown adipocytes and found that more mitochondria surrounded LDs and lipid synthesis was enhanced to promote the expansion of LDs [22]. In our study, we found that the overexpression of *PLIN5* increased the number of cellular LDs, whereas *PLIN5* knockdown decreased the number of cellular LDs (Figure 4C,D). Moreover, we also found that *PLIN5* overexpression promoted LD contact with mitochondria.

It is well known that organelle contacts usually induce the exchange of proteins in the outer membrane. LD is a highly dynamic organelle, which contacts other organelles frequently, such as endoplasmic reticulum (ER), mitochondria, peroxisome, and autolysosome. Many studies have reported that LD–ER contacts resulted in ER proteins, such as lipid synthetases (*DGAT2*, *GPAT4*) transferring to LDs [39,40,41,42]. LD contact with ER is important to LDs’ expansion and cellular lipid homeostasis. A previous study showed an interesting phenotype involving LDs contacting mitochondria and clearing harmful proteins from the outer mitochondrial membrane [26]. A high level of cellular ROS induced the damage, and when accumulated damage exceeded a certain threshold, the cells would undergo the apoptosis process. During this process, some specific proteins were translocated to mitochondria such as pro- and anti-apoptotic proteins, for example, *BAX, BCL-XS, BIK, BAK, BCL-2, BCL-XL,* and *CED* [43,44]. Among these proteins, *BAX* played an important role in leading to a permeabilization of the outer mitochondrial membrane, which was able to subsequently induce the release of cytochrome c and apoptosis [45,46]. Interestingly, *BAX* and *BCL-XL* contained a protein domain consisting of two α-helices, which allowed them to localize to LDs. When LDs were in contact with mitochondria, *BAX* and *BCL-XL* were translocated to LDs from mitochondria [26]. Therefore, we considered that enhancing LD contact with mitochondria promoted the translocation process, which would subsequently modulate the stress response. In the present study, *PLIN5* overexpression enhanced the contacts between LDs and mitochondria, and the cellular ROS levels were significantly decreased (*p* < 0.05; Figure 3 and Figure 4). Moreover, we also detected the influence of *PLIN5* expression on the expression of several anti-oxidant genes. The results showed that *PLIN5* did affect several anti-oxidant enzymes. We considered that there was a little effect, because not so many anti-oxidant enzymes such as some isoforms of *SOD, CAT,* and *GPX* can be influenced by the change of *PLIN5* expression. Therefore, the *PLIN5*-mediated LD contact with mitochondria could be an important mechanism for cells to respond to oxidative stress. For further study, the proteins of mitochondria and LDs in cells with *PLIN5* overexpression and control could be isolated, respectively, to investigate whether *PLIN5* overexpression promotes the proteins’ translocation between these two organelles and to analyze the terms of proteins translocated through the mass spectrum method.

We investigated the regulatory pathway of *PLIN5* during the oxidative stress process. It is well known that the JNK-p38 signaling pathway plays an important role in the stress response. When the ROS levels (such as cellular hydrogen peroxide) were elevated, apoptosis signal-regulating kinase 1 (*ASK1*) was activated and subsequently sustained the activation of *JNK* and *p38 MAPK* signaling [47]. The activated *JNK* and *p38 MAPK* signaling would further activate ATFs’ expression [31,32,33,34]. In our study, JNK-p38 MAPK signaling was activated by hydrogen peroxide treatment, and then *ATF1* and *ATF4* expression levels were significantly increased (*p* < 0.05; Figure 6A), which corresponds to the previous studies. Through bioinformatic analysis, we found that the promoter region of *PLIN5* contained the binding sites of ATFs. Therefore, we considered that the expression of ATFs could affect the expression levels of *PLIN5*. Overexpression of *ATF1* or *ATF4* indeed up-regulated *PLIN5* (Figure 6B,C). Furthermore, we also validated the regulatory role of ATFs on *PLIN5* expression experimentally, through dual luciferase reporter gene assay. The results confirmed that ATFs did indeed enhance the transcriptional activity of *PLIN5*. Therefore, we demonstrated that the ROS-JNK-p38-ATFs regulatory axis modulated the expression of *PLIN5* so that it regulated the cellular stress response process. Moreover, many studies have reported that *ASK1* signaling played an important role in NAFLD/NASH processes by promoting the inhibition of lipid and glucose metabolism [48,49,50] and by driving a strong inflammatory response [51]. Currently, *ASK1* has become a key therapeutic target for NAFLD/NASH. For example, Selonsertib (GS-4997) is a highly selective and potent *ASK1* inhibitor with potential anti-inflammatory, anti-tumor, and anti-fibrotic activities [52]. ASK1-JNK-p38 signaling was activated in NAFLD/NASH; therefore, *PLIN5* expression levels were supported to increase also. Our results showed that *PLIN5* expression was indeed up-regulated in liver tissues of mice fed with MCDD, which supported our hypothesis. We considered that *PLIN5* up-regulation could be a rescue mechanism during NAFLD/NASH processes. Increased *PLIN5* expression promoted LDs contacting mitochondria, enhanced the expression of mitochondrial functional genes and subsequently alleviated the cellular oxidative stress. We found that many kinds of tumors cells showed low expression levels of *PLIN5* (Figure 7A). Previous studies showed that NAFLD and NASH were well-known risk factors of hepatocellular carcinoma (HCC) [53,54], whereas and HCC was a lipid-rich tumor. Patients with obesity and NAFLD/NASH show an increased intake of dietary fatty acids (FAs). Meanwhile, insulin resistance enhances lipolysis of adipose tissue, which causes an increased exogenous FA supply and results in the development of a “lipid-rich” environment for hepatocytes. As we all know, more FAs would promote cells to generate more ROS through β-oxidation process. High level of cellular ROS often induced cellular stress and promoted cell apoptosis. However, we found that *PLIN5* could reduce cellular ROS levels and reduce cell apoptosis in the present study. Moreover, we also found that expression of *PLIN5* could increase cellular lipid content. Previous studies reported that the lipid-rich environment is considered to promote the proliferation and metastasis of tumor cells [55,56,57]. Therefore, we considered that both functions of *PLIN5*, regulating cellular ROS levels and regulating cellular lipid content and lipolysis, could influence the tumor development process. Consequently, the down-regulation of *PLIN5* could be a predisposition for tumors’ occurrence. *PLIN5* can be a good therapeutic target for NAFLD due to its ability to protect against oxidative stress and enhance mitochondrial function.

In the present study, we found that increase of cellular ROS induced by hydrogen peroxide or LPS treatment could up-regulate *PLIN5* expression. We then identified that ROS regulates the expression levels of *PLIN5* through JNK-p38-ATF signaling. Furthermore, we found that *PLIN5* could regulate the expression levels of mitochondrial cytochrome c oxidases (COXs) such as *COX2*, *COX4* and *CS*. Therefore, *PLIN5* could decrease cellular ROS levels through reducing the generation of ROS products by mitochondria, because up-regulation of COXs could reduce ROS products. Above is the novelty of this study. However, there are also several limitations in this study. The regulatory mechanism of *PLIN5* modulating the expression of COXs need further study. For example, studying the mechanism of protein exchange between LD and mitochondria during these two organelles contact. The LDs in cells with *PLIN5* overexpression could be isolated and the LD-related proteins on LD surface could be analyzed by mass spectrometry. Subsequently, whether *PLIN5* could promote protein exchange between LD and mitochondria can be investigated, by detecting the levels of mitochondrial-derived proteins on LD surface. Moreover, we noted that *PLIN5* could influence the expression levels of cellular anti-oxidative enzymes, such as *SOD1, SOD2, GPX1, GPX2,* and *CAT*. Although the effect of *PLIN5* on the expression of these enzymes was very mild, the mechanism is worth further study.

**Figure 8:**
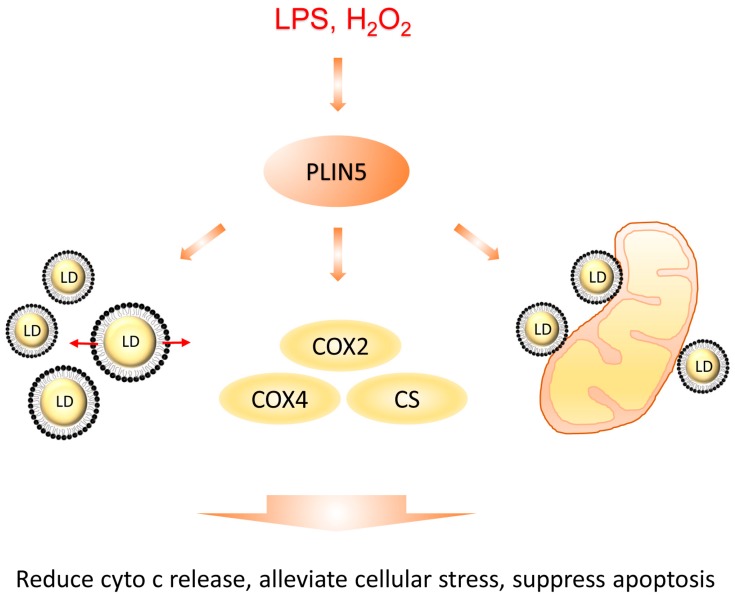
Diagrammatic sketch of this study. *PLIN5* was up-regulated by cellular stress induced by H_2_O_2_ or LPS treatment. Then, the increased *PLIN5* levels promoted cellular LD formation and expansion, expression levels of COXs and LDs contacting with mitochondria. Subsequently, LD formation and expansion reduced the levels of cellular fatty acids, which promoted the alleviation of stress. COXs’ up-regulation reduced the release of cytochrome c from mitochondria to cytoplasm and reduced the mitochondrial damage. The contacts between LDs and mitochondria helped the transfer of potential harmful proteins from mitochondria to LDs. Therefore, cellular stress was alleviated.

In conclusion, ROS-mediated activation of JNK-p38-ATF signaling up-regulated expression levels of *PLIN5*, and, then, increased *PLIN5* levels enhanced lipid synthesis and promoted LD contact with mitochondria, which helped cells to modulate stress response (Figure 8). Moreover, our study suggests that *PLIN5* could be a therapeutic target for NAFLD.

## 4. Materials and Methods

### 4.1. Animals

Six-week-old c57/bl6 male mice were purchased from Hubei Center for Disease Control and Prevention. All mice were housed in a normal environment and were provided with food and water. The methods were carried out in accordance with approved guidelines from Huazhong Agricultural University and the scientific, ethical, and legal principles of the Hubei Regulations for the Administration of Affairs Concerning Experimental Animals. All of the experimental protocols were subject to approval by the Ethics Committee of Huazhong Agricultural University (HZAUMU2013-0005). The mice were fed with either a methionine-choline-deficient diet (MCDD) or a high-fat diet (HFD) to make a NAFLD phenotype in liver tissue. The mice were divided into nine groups, which were fed with MCDD for 0 week (control groups), 1 week, 2 weeks, 3 weeks, 4 weeks, 6 weeks, 8 weeks, and HFD for 10 weeks. The control groups of mice were fed with chow diet.

### 4.2. Cell Culture

The HepG2 cell line was gifted by the lab of Prof. Xianghua Yan, Huazhong Agricultural University (Wuhan, China), which was purchased from the Type Culture Collection of the Chinese Academy of Sciences (Wuhan, China). HepG2 cells were cultured in Dulbecco’s Modified Eagle Medium (DMEM; HyClone, Logan, UT, USA) with 10% fetal bovine serum (FBS; #SH30396.03, Hyclone, Canada), 100 unit/mL penicillin, and 100 μg/mL streptomycin in dishes at 37 °C, in a humidified atmosphere, with 5% CO_2_. For oleic acid treatment, a 20 mM oleic acid-phosphate buffer saline (PBS) mixture and 20% FA-free bovine serum albumin (BSA) medium were prepared, and both media were heated in a 70 °C water bath for 30 min. Finally, the media were mixed. The 10 mM oleic acid-BSA mixture was added to the cell cultural medium at 1:49 (*v:v*). The cells were then either seeded on slides, or on plates that had been washed three times using PBS. Then, 1 mL oleic acid medium was added to the well, and the cells were cultured for 12 h.

### 4.3. Antibodies

Rabbit polyclonal antibodies that were used included anti-PLIN5 (#26951-1-AP, Proteintech, Wuhan, China), anti-ATF1 (#11946-1-AP, Proteintech, Wuhan, China), anti-ATF4 (#10835-1-AP, Proteintech, Wuhan, China), anti-MAPK14 (p38; #A10832, Abclonal, Wuhan, China), anti-Phospho-MAPK14-T180/Y182 (#AP0526, Abclonal, Wuhan, China), anti-Phospho-JNK1/2/3- T183/T183/T221 (#AP0631, Abclonal, Wuhan, China), anti-ASK1 (#A6274; rabbit polyclonal antibody; ABclonal; 1:2000 dilution), anti- p-ASK1 (#AP0394; rabbit polyclonal antibody; ABclonal; 1:2000 dilution), anti-VDAC1/Porin (#55259-1-AP, Proteintech, Wuhan, China), and anti-GAPDH (#AC027, Abclonal, Wuhan, China). The mouse monoclonal antibody that was used included anti-JNK1/2/3 (#A11119, Abclonal, Wuhan, China). The following secondary antibodies were used: HRP (horseradish peroxidase)-labeled Goat Anti-Rabbit IgG (H+L; #AS014, Abclonal, Wuhan, China), and HRP-labeled Goat Anti-Mouse IgG (H+L; #AS003, Abclonal, Wuhan, China).

### 4.4. Transfection Assay

Cells were seeded on a 6-well plate or on slides in a 24-well plate. Then, the cells were transfected with Lipo8000™ Transfection Reagent (#C0533, Beyotime, Nanjing, China). For the preparation of RNAi working solution, 10 μL siRNA oligo (20 μM, Ribobio, Guangzhou, China) was mixed with 10 μL Lipo8000 regent in 100 μL DMEM. For the preparation of the overexpression working solution, 2.5 μg plasmid was mixed with 4 μL Lipo8000 regent in 50 μL DMEM. The working solution was added in the plate well and incubated for 6 h. Then, the plate well was changed with fresh cultural medium (DMEM with 10% FBS) for another 48 h of culture.

### 4.5. Plasmid DNA Construction

For the overexpression assay and the localization assay, expression vector and fluorescence-labeled vector were constructed. In brief, the *PLIN5/ATF1/ATF4* CDS region was amplified by the cDNA library of HepG2 cells using KOD-Plus-Neo DNA polymerase (#KOD-401, TOYOBO, Shanghai, China). After gel extraction, the *PLIN5* CDS fragment was cloned into the digested pcDNA3.1 vector (digestion sites, HindIII and BamHI) using a seamless cloning kit (#C112-01, ClonExpress II One Step Cloning Kit, Vazyme, Nanjing, China). For the localization assay, the gene CDS region was cloned into the digested pCMV-C-Dsred (#D2624, Beyotime Biotechnology, Nanjing, China) or pCMV-C-EGFP (#D2626, Beyotime Biotechnology, Nanjing, China). For the luciferase reporter assay, the promoter region of *PLIN5* (about 2000 bp upstream of the transcription initiation site of *PLIN5*) was cloned into the digested pGL3-basic vector (digestion sites, KpnI and XhoI).

### 4.6. Hydrogen Peroxide and LPS Treatment

The treatment process was the same as in our previous study [12]. Briefly, 30% hydrogen peroxide (i.e., 10 M) was diluted 10,000× by DMEM medium to 1 mM concentration, after the medium had been sterilized using a 0.22 μm filter. The hydrogen peroxide was then diluted to 200 μM and the medium was used to treat cells. The cells were then washed three times using PBS and were treated with different concentrations of hydrogen peroxide media; this operation is important for the treatment of hydrogen peroxide, especially if in low concentrations. Due to the significant impacts of small amounts of metals, such as iron and copper, on the outcomes of in vitro experiments, the medium contained ferric nitrate·9H_2_O (0.1 mg/L). No other iron or copper was present. The water that was used in this experiment was double distilled and deionized.

### 4.7. Lipid Droplets Marking and Observation

The cell slides were fixed with 4% paraformaldehyde for 15 min at room temperature. The slides were stained with BODIPY 493/503 (#D3922, Invitrogen, Carlsbad, CA, USA) for 10 min at 37 °C and were then stained with DAPI (#G-1012, Servicebio) for 10 min at 37 °C. After washing three times with PBS for 10 min each, the slides were sealed with an anti-fluorescent quenching solution (#P36961, ProLong™ Diamond Antifade Mountant, Invitrogen, Thermo Fisher, USA) for confocal microscopic observation (63× oil lens, BODIPY FL and DAPI channels, Zeiss LSM 800, Germany).

### 4.8. Western Blot

Western blotting was performed as reported previously [58]. Briefly, cells were collected and homogenized in lysis buffer (#P0013, Beyotime Biotechnology, Nanjing, China). Then, the homogenates were incubated with an SDS-PAGE sample loading buffer (#P0015A, Beyotime Biotechnology, Nanjing, China) at 98 °C for 10 min. Subsequently, the samples were separated by 10% sodium dodecyl sulfate-polyacrylamide gel electrophoresis (SDS-PAGE) and were transferred to a polyvinylidene fluoride (PVDF) membrane (Biorad, USA) using a semidry electrophoretic apparatus. The blocked membranes (#P0252-100mL, QuickBlock™ Blocking Buffer for Western Blot, Beyotime Biotechnology, Nanjing, China) were incubated with antibodies overnight at 4 °C. The blots were extensively washed three times with tris-buffered saline with tween20 (TBST) buffer for 10 min and were incubated under gentle agitation with the primary antibodies for immunodetection at 37 °C for 1.5 h (diluted in QuickBlock™ Primary Antibody Dilution Buffer for Western Blot, #P0256, Beyotime Biotechnology, Nanjing, China). Then, the blots were extensively washed three times with TBST. Subsequently, blots were incubated under gentle agitation with the secondary antibodies for immunodetection at 37 °C for 1 h (diluted in QuickBlock™ Secondary Antibody Dilution Buffer for Western Blot, #P0258, Beyotime Biotechnology, Nanjing, China). For detection, M5 eECL Western Blot Kit (#MF-078-01, Mei5bio, Beijing, China) and the chemiluminescence imaging system (LAS4000, ImageQuant, Germany) were used.

### 4.9. Real-Time PCR

Real-time PCR was performed using the QuantStudio 6 Flex Real-Time PCR System (ABI, Thermo Fisher, Shanghai, China) and Roche LightCycler^®^ 480 (Roche, Switzerland), and the following PCR program: Denaturation at 95 °C for 10 min; amplification for 45 cycles at 95 °C for 15 s; annealing and extension at 60 °C for 1 min. 2× SYBR Green qPCR Master Mix (#B21203, Bimake, Shanghai, China) were used for the detection of RT-qPCR. Primer sequences are shown in Table 1. Specific amplifications for certain PCR reactions were assessed using a melting curve. One negative control reaction, in which the cDNA template was replaced by water, was performed to avoid potential contamination. The sample from each well was repeated three times, and the comparative Ct (2^−ΔΔCt^) value method was used for relative quantification. GAPDH (NM_002046.6) was used as the reference gene.

### 4.10. Apoptosis and Mitochondrial Membrane Potential Analysis

The analysis was performed by Servicebio Co., Ltd. (Wuhan, China). Briefly, the cells with transfections were collected through trypsin digestion. Then, the cells were incubated by annexin V-FITC and propidium iodide (PI). Then, the apoptosis rates were detected through flow cytometry (Ex = 488 nm, FL1 (Em = 525 ± 20 nm) and FL2 (Em = 585 ± 21 nm)). Flow Jo software was used to analysis the rates of cells in different conditions. For mitochondrial membrane potential (MMP) analysis, JC-1 probe was used. Briefly, the cells were collected and counted. The cells were then incubated with 10 μg/mL JC-1 probe at 37 °C for 20 min. Cells were detected through flow cytometry (Ex = 488 nm, FL1 (Em = 525 ± 20 nm) and FL2 (Em = 585 ± 20 nm)). Flow Jo software was used for the analysis.

### 4.11. Bioinformatics and Data Analysis

The survival predication was performed using the GEPIA database (http://gepia.cancerpku.cn/). The prognosis analysis and gene expression analysis were performed according to the construction of the creator of this database [59].

### 4.12. Dual-Luciferase Reporter Assay

The promoter region of *PLIN5* was amplified by PCR with total DNA of HepG2 cells. The primers were used as following, F: 5′-GAAAACTGGATCGGATGAATTGG-3′ and R: 5′-CACCCCCGCCGGTCCCGC-3′. Then, the promoter region was cloned into the pGL3-basic vector. Then, the reconstructed vector was co-transfected with the ATFs expression or pcDNA3.1 (control) vectors and TK vector into HepG2 cells seeded into the 12-well plate. Moreover, pGL3-basic vector was used as the negative control and pGL3-CMV vector was used as the positive vector. Luciferase enzymatic activity was measured by a microplate reader from a multi-wavelength measurement system (PE Enspire, PerkinElmer, Germany) using a dual-luciferase reporter assay system (#RG027, Beyotime Biotechnology, Nanjing, China). The relative light unit (RLU) was normalized by a control group. The result was showed by the relative RLU. All transfections were performed in triplicate, and the data are expressed as the means ± SD.

### 4.13. Immunohistochemistry Assay

Liver tissue samples of MCDD- and HFD-fed mice and control mice were collected. Then, the samples were fixed in 4% paraformaldehyde for 24 h. The immunohistochemistry assay was entrusted by Servicebio (Wuhan, China). The detailed processes of this experiment can be referred to in our previous study [12].

### 4.14. Isolation of Cytosolic and Mitochondrial Fractions

The cell mitochondria isolation kit (#C3601, Beyotime Biotechnology, Nanjing, China) was utilized to isolate the cytosolic and mitochondrial fractions. Briefly, wash the cells with cold PBS and harvest the cells by trypsin-EDTA solution. Re-wash the cells two times and collect the cells by centrifuge, and then remove the supernatant. Add 1 mL mitochondrial isolation regent (with 1 mM PMSF) and resuspend the cells, and then incubate the suspension in an ice bath. Then the cell suspension was transferred to a glass homogenizer of appropriate size, and the homogenate was about 10–30 times. Centrifuge the cell homogenate at 600× *g*, 4 °C for 10 min. Then carefully transfer the supernatant to another centrifugal tube and centrifuge for 10 min at 11,000× *g*, 4 °C. The precipitation was the isolated mitochondria. The supernatant collected was then centrifuged for 10 min at 12,000× *g*, 4 °C. The supernatant was the cytoplasmic protein without mitochondria.

### 4.15. Fluorescence Image Analysis

The ImageJ software was utilized to analyze the number of mitochondria interacting LDs. Briefly, the fluorescence intensity was analyzed along with the dotted line (Figure 4G). If the LD is contacting mitochondria, the signal of Dsred (mitochondria) can be detected between or overlap the signal of BODIPY493/503 (lipid droplet). If the LD does not contact mitochondria, the signal of Dsred (mitochondria) is supposed to be losing between the signal of BODIPY493/503 (lipid droplet).

### 4.16. Survival Analysis and Normal/Cancer Gene Expression Comparison Analysis

The survival predication was performed by the GEPIA database (http://gepia.cancer-pku.cn/). The prognosis analysis and gene expression analysis were performed according to the construction of the creator of this database [59].

### 4.17. Statistical Analyses

All quantitative experiments were evaluated for statistical significance using the software GraphPad Prism v.5.0 (GraphPad Software, Inc. 7825 Fay Avenue, Suite 230 La Jolla, CA, USA), after verifying the normality of values and equivalence of variances. For lipid droplet counts, pixel quantification, LD-mitochondria contact site counts, fluorescence intensity, and qPCR analyses, means ± s.d. are displayed, and the statistical differences between overexpression or RNAi-treated or peroxide hydrogen-treated samples and controls were addressed using Student’s two-tailed *t*-tests. The Student’s *t*-test was utilized because the sample size in the experiment was small, and a sample mean and standard deviation can be obtained, and additionally samples came from normal or approximate normal population. A *p*-value < 0.05 was considered statistically significant.

## Figures and Tables

**Table 1:** Primer used for SYBR Green I qRT-PCR validation.

Gene Symbol	Primer Sequence 5′-3′
***PLIN5 (Perilipin 5)***	Forward: AAGGCCCTGAAGTGGGTTC
	Reverse: GCATGTGGTCTATCAGCTCCA
***CS***	Forward: GCTCCTGTTTCCATGGGTCA
	Reverse: TGCCAAAGCATGTCCAGCTA
***COX2***	Forward: GCTGTCCCCACATTAGGCTT
	Reverse: ACCGTAGTATACCCCCGGTC
***COX4***	Forward: CCCGGCATTTTACGACGTTC
	Reverse: AAAAATGTACACCTGCCGCC
***ATF1***	Forward: TTCGGATCTACCTGGGAGGG
	Reverse: CTGATAAAGATGATACCTGTTGAGC
***ATF3***	Forward: GACCAACCATGCCTTGAGGA
	Reverse: GGATGGCAAACCTCAGCTCT
***ATF4***	Forward: TAAGCCATGGCGTGAGTACC
	Reverse: GCGCTCGTTAAATCGCTTCC
***GAPDH***	Forward: CTGGGCTACACTGAGCACC
	Reverse: AAGTGGTCGTTGAGGGCAATG
***CAT***	Forward: TGGGATCTCGTTGGAAATAACAC
	Reverse: TCAGGACGTAGGCTCCAGAAG
***GPX1***	Forward: CAGTCGGTGTATGCCTTCTCG
	Reverse: GAGGGACGCCACATTCTCG
***GPX2***	Forward: GAATGGGCAGAACGAGCATC
	Reverse: CCGGCCCTATGAGGAACTTC
***SOD1***	Forward: GGTGGGCCAAAGGATGAAGAG
	Reverse: CCACAAGCCAAACGACTTCC
***SOD2***	Forward: TTTCAATAAGGAACGGGGACAC
	Reverse: GTGCTCCCACACATCAATCC
***TXNRD1***	Forward: ATGGGCAATTTATTGGTCCTCAC
	Reverse: CCCAAGTAACGTGGTCTTTCAC
***PRDX3***	Forward: ACTGTGAAGTTGTCGCAGTCT
	Reverse: CACACCGTAGTCTCGGGAAA

## Abbreviations

AMPK	protein kinase AMP-activated catalytic subunit
ASK	apoptosis signal-regulating kinase
ATF	activating transcription factor
BODIPY	4,4-difluoro-1,3,5,7,8-pentamethyl-4-bora-3a,4a-diaza-s-indacene
CAT	catalase
COX	cytochrome c oxidase subunit IV
CS	citrate synthase
Cyto c	cytochrome c
DCFH-DA	2,7-Dichlorodihydrofluorescein diacetate
GEPIA	gene expression profiling interactive analysis
GPX	glutathione peroxidase
HFD	high-fat diet
JC-1	5,5′,6,6′-Tetrachloro-1,1′,3,3′-tetraethyl-imidacarbocyanine
JNK	jnk c-Jun N-terminal kinase
LD	lipid droplet
LIHC	liver hepatocellular carcinoma
LPS	lipopolysaccharide
MAPK	mitogen-activated protein kinase
MCDD	methionine-choline-deficient diet
NAFLD	non-alcoholic fatty liver disease
NASH	non-alcoholic hepatitis
P38	mitogen-activated protein kinase 14
PAT	perilipin, adipophilin, and TIP47
PLIN5	perilipin 5
PNPLA2/ATGL	patatin like phospholipase domain containing 2
ROS	reactive oxygen species
SOD	superoxide dismutase
